# Formulation Effects on the Mechano-Physical Properties of In Situ-Forming Resilient Hydrogels for Breast Tissue Regeneration

**DOI:** 10.3390/jfb15070176

**Published:** 2024-06-28

**Authors:** Daniella Goder Orbach, Meital Zilberman

**Affiliations:** Department of Biomedical Engineering, Faculty of Engineering, Tel-Aviv University, Tel-Aviv 69978, Israel; goderdan@mail.tau.ac.il

**Keywords:** gelatin, resilience, porous hydrogel, adipose tissue regeneration

## Abstract

The need for a long-term solution for filling the defects created during partial mastectomies due to breast cancer diagnosis has not been met to date. All available defect-filling methods are non-permanent and necessitate repeat procedures. Here, we report on novel injectable porous hydrogel structures based on the natural polymers gelatin and alginate, which are designed to serve for breast reconstruction and regeneration following partial mastectomy. The effects of the formulation parameters on the mechanical and physical properties were thoroughly studied. The modulus in compression and tension were in the range of native breast tissue. Both increased with the increase in the crosslinker concentration and the polymer–air ratio. Resilience was very high, above 93% for most studied formulations, allowing the scaffold to be continuously deformed without changing its shape. The combination of high resilience and low elastic modulus is favored for adipose tissue regeneration. The physical properties of gelation time and water uptake are controllable and are affected mainly by the alginate and N-(3-dimethylaminopropyl)-N’-ethylcarbodiimide hydrochloride (EDC) concentrations and less by the polymer–air ratio. In vitro cell viability tests were performed on mouse preadipocytes and indicated high biocompatibility. The minimally invasive nature of this approach, along with the excellent properties of the scaffold, will enable the filling of complex voids while simultaneously decreasing surgical costs and greatly improving patient well-being.

## 1. Introduction

Breast cancer treatment usually begins with a full or partial mastectomy, after which reconstruction is offered. Currently available reconstruction options, such as silicone implants, entail many possible complications and require repeat procedures. These drawbacks have led to the search for a better solution. Fat grafting has been attempted, but the fat undergoes up to 70% resorption [1,2]. Over the past several years, surgeons have turned to products indicated for other purposes, such as facial fillers. Hyaluronic acid, for example, temporarily improves the esthetic outcome but undergoes significant resorption and requires constant upkeep. The need for breast tissue regeneration as an alternative to synthetic implants is also derived from changes undergone by the human body over time: aging, weight changes and glandular activity alter the stiffness of the breast, and an implant that once fit may eventually become inappropriate [3]. Efforts have, therefore, been made in recent years to develop a breast implant or filler that can act as a scaffold for breast tissue regeneration.

A complete mastectomy is not required when the breast tumor is small enough, and a lumpectomy is suggested. In this case, only the defect site needs to be refilled rather than replacing the entire breast. Because autologous fat is resorbed over time, using it to fill the defects requires repeat procedures. To overcome this, it is hypothesized that the addition of a scaffold may present a suitable environment for cells to adhere and proliferate [4,5]. The female breast is composed of glandular, fibrous and adipose tissue [3,6]. In this research, we focused on adipose tissue regeneration, as it allows filling the defect created by the lumpectomy and regaining the original breast shape. Fortunately, adipose tissue in the breast has a naturally high concentration of adipose-derived stem cells (ADSCs), which can be utilized for new tissue growth in vivo. However, the scaffold must fulfill several requirements so that native ADSCs will be able to adhere to the scaffold and then differentiate specifically into adipose tissue.

Mechanically, the implant must possess properties similar to native tissue. Otherwise, the implant may fail, or the surrounding tissue may be harmed even further [5]. Matrix elasticity has been found to directly affect stem cell differentiation [4,7]. The use of soft and elastic materials is therefore necessary in order to develop a mechanically compatible functional breast implant [6,8,9]. The native stiffness of adipose tissue is approximately 2 kPa [10,11], and it was reported that ADSCs cultured on a matrix of this stiffness upregulated adipogenic markers, which were reduced when substrate stiffness was increased [10]. It was also found that if the matrix is too stiff, cellular activities decrease due to a disturbance in cell movement, whereas if it is too soft, cell attachment and proliferation are difficult [12,13]. In another study that investigated in vitro adipocyte differentiation on polyacrylamide gel, it was found that a stiffness higher than 4.1 kPa resulted in high adipocyte spreading, while lower stiffness resulted in a more spherical phenotype [14]. As mentioned, recent tissue engineering research has focused on finding scaffolds that mimic natural tissue behavior and promote cell differentiation into various soft tissues. Scaffolds used as alternatives for muscle, cartilage, bone and brain tissues have been widely studied, but their stiffnesses are either too low or too high for fat tissue differentiation [15,16].

Another mechanical aspect that must be addressed is scaffold resilience. Resilience is the ability of a material to deform elastically without energy loss [17]. A cyclic load is applied to the breast when performing any type of daily activity, such as walking, running, and jumping. Forces that cause it to deform are applied to the breast even when lying down statically, and it needs to regain its shape after the load is removed [6]. Implants must be able to repeatedly deform elastically while maintaining their shape. They must, therefore, have high resilience.

Scaffold porosity is another important characteristic in tissue engineering, not only because it helps modify the mechanical properties but also for the implant’s functionality. Porous hydrogels have shown promising results in applications such as skin [18], cardiac muscle [19] and cartilage [20] engineering. In adipose tissue regeneration, the scaffold should have interconnected pores larger than 100 µm to enable sufficient flow of nutrients and oxygen while ensuring abundant cellular infiltration [2].

Most research in this area has concentrated on the use of synthetic polymers for creating structures to which adipose cells can adhere, creating an environment for these cells to thrive. Examples of recent research on hydrogels that support adipocyte growth are a polyethylene glycol hydrogel incorporated with adipose extracellular matrix [21], a methacrylamide-modified gelatin and methacrylated ĸ-carrageenan hydrogel [22] and a poly (L-lactide-co-ε-caprolactone) copolymer [23]. However, synthetic polymers are not ideal for biomedical applications due to their lower biocompatibility and biofunctionality compared to natural polymers [24]. Synthetic polymers have acidic degradation products that cause strong inflammatory responses upon scaffold degradation, whereas the natural polymers’ degradation process is highly biocompatible. Furthermore, synthetic polymers do not promote cell adhesion as well as natural polymers, leading to resorption of de novo tissue over time. None of the above examples exhibit the combination of high resilience and elasticity necessary for the thriving of preadipocytes.

In this paper, we describe a novel injectable natural polymer-based scaffold for breast tissue regeneration that is soft, highly resilient, and biocompatible. The scaffold is based on polymers that are widely used for biomedical applications due to their high biocompatibility: gelatin, a derivative of collagen, which is the most commonly occurring protein in the extracellular matrix (ECM), and alginate, a natural polysaccharide derived from brown algae that is commonly used in biomedical applications due to its high biocompatibility and versatility [4,25,26]. Although gelatin can form physically crosslinked hydrogels, these do not exhibit the properties required for a breast tissue regeneration scaffold [4]. A chemical crosslinking agent is, therefore, added to the formulations. The crosslinking agent chosen for this study is N-ethyl-N-(3-dimethylaminopropyl) carbodiimide (EDC). Crosslinking between gelatin and alginate chains using EDC alters the scaffold’s properties and enables obtaining a variety of property ranges. 

The hydrogel described in the current study can be foamed prior to application, making it porous so that cells can successfully migrate and proliferate into the scaffold. After foaming, the reported scaffold is applied to the defect area, where it crosslinks in situ, allowing it to gain the exact shape of the defect. This implant is designed to act as a temporary filler and scaffold for new tissue growth and adipose tissue regeneration, improving patient care and reconstruction outcomes. The current article focuses on the effects of the formulation parameters on the mechanical and physical properties of this scaffold. A cell viability study is also reported.

## 2. Materials and Methods

Hydrogels were prepared from coldwater fish skin gelatin (G7041, Sigma-Aldrich, Rehovot, Israel), alginic acid sodium salt (A1112, Sigma-Aldrich, Rehovot, Israel) and N-(3-dimethylaminopropyl)-N’-ethylcarbodiimide hydrochloride (EDC) (E7750, Sigma-Aldrich, Rehovot, Israel). Their crosslinking degree was tested using a ninhydrin assay purchased from Sigma-Aldrich, Rehovot, Israel.

Biological evaluation was performed using Mouse embryonic 3T3-L1 preadipocytes obtained from American Type Culture Collection, Manassas, VA, USA. Modified Eagle’s Medium (MEM) supplemented with fetal bovine serum, L-glutamine and penicillin–streptomycin–nystatin were all purchased from Biological Industries. The AlamarBlue™ assay was purchased from Invitrogen™ (Rhenium, Modi’in, Israel). 

### 2.1. Hydrogel Preparation

**Figure 1:**
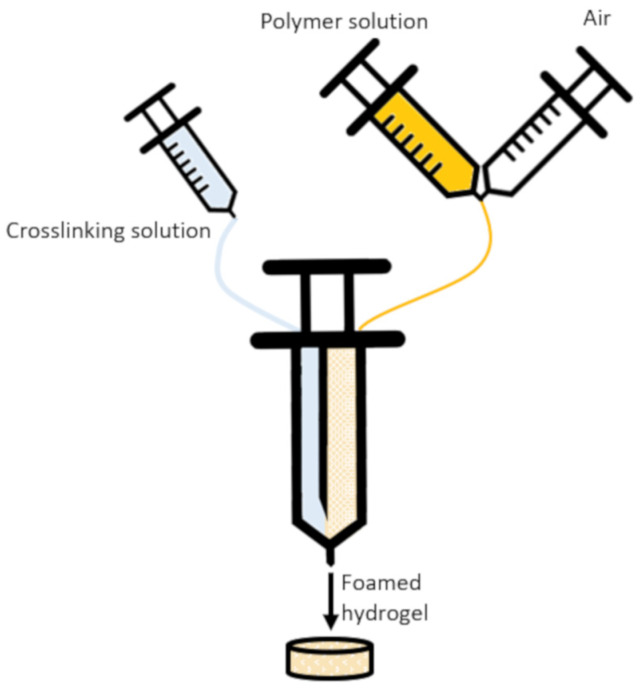
An illustration showing the foaming process and preparation of hydrogel samples. A double-barrel syringe is loaded with a crosslinking solution on one side and a foamed polymeric solution on the other side. The foamed polymeric solution is prepared by mixing polymer and air. The contents of the syringe are then emptied through a static mixer into a mold, and the foamed hydrogel is formed.

Polymer solution was prepared by dissolving gelatin and alginate in double-distilled water (DDW) at 60 °C, and a crosslinking solution was prepared by mixing various EDC concentrations in DDW. The two solutions were loaded into a commercial double syringe fitted with a static mixer tip (Mixpac™, L-system 2.5 mL, 4:1 volume ratio, purchased from Sulzer Mixpac, AG, Haag, Switzerland), which homogeneously mixes the two solutions as the syringe contents are pushed out. To create porous hydrogels, an extra step was added prior to loading the double syringe: the polymer solution was loaded into a regular syringe, and a second syringe was loaded with air. Using a 3-way stopcock, the polymer solution and air were vigorously mixed until the air was fully mixed into the polymer solution. The resulting polymer–air mixture was then loaded into the double syringe, and the process continued as described above. An illustration of the process is presented in Figure 1.

When foamed, the concentration of the polymeric solution must be adjusted to account for the dilution factor introduced by the addition of a constant volume of EDC solution. This was found to be a factor of the ratio of polymer to the total polymer and air volume. Polymer and crosslinker content in each solution were calculated using Formulas (1) and (2), where $W_{pol}$ and $W_{EDC}$ are the weights of polymer and crosslinker in the solution, respectively, $V_{sol}$ is the solution volume, $C_{pol}$ and $C_{EDC}$ are the polymer and crosslinker concentrations in the final solution, respectively, and p and a are the polymer and air parts, respectively, from the polymer–air foaming ratio.

(1) $$W_{pol}=V_{sol}\cdot C_{pol}\left(1+\frac{p+a}{4p}\right)$$

(2) $$W_{EDC}=V_{sol}\cdot C_{EDC}\left(1+\frac{4p}{p+a}\right)$$

A series of hydrogels were prepared with a gelatin concentration of 200 mg/mL and various concentrations of alginate and EDC. The formulations and foaming ratios are presented in Table 1.

### 2.2. Mechanical Properties

Mechanical tests were performed using a 5500 Instron Universal Testing machine (Instron, Norwood, MA, USA), model number 5944, with a 10 N load cell. Cylindrical samples (12 mm diameter, 8 mm height) were prepared by casting the hydrogel into silicone molds. The cylinders were taken out of the mold after crosslinking occurred. 

#### 2.2.1. Compression

A stress–strain curve was obtained for each formulation by compressing the hydrogel samples at a constant rate of 5 mm/min. The test ended when the sample was compressed to 90% strain or a force of 9 N (load cell limit). The compression modulus was calculated from the linear part of the stress–strain curve between 10 and 30% strain. Maximum force was also recorded, though none of the samples failed during testing. 

Compression tests were also performed after water uptake. Samples were immersed in DDW for 24 h at 37 °C in an incubator. Since the hydrogel swells in water, sample dimensions after swelling were measured using a caliper, and a compression test was performed as described above. At least six repetitions were performed for each formulation.

#### 2.2.2. Tension

The hydrogel’s characteristics were tested in tension as well, using the above-described testing system. Dog bone-shaped samples were pulled in tension at a rate of 5 mm/min until failure occurred, and Young’s modulus was calculated from the linear part of the stress–strain curve. At least four repetitions were performed for each formulation.

#### 2.2.3. Resilience

The resilience of the hydrogels was tested by subjecting the samples to 50 cycles of compression to 40% strain and release to the original height at a rate of 5 mm/min. Resilience was measured using the following formula:

(3) $$Resilience\left(\%\right)=\frac{L-U}{L}\times 100$$

where L is the area under the loading stress–strain curve, which indicates the energy required for deformation, and U is the area under the unloading stress–strain curve, which indicates the release of stored energy. L − U is the hysteresis loop, indicating energy dissipation. At least five repetitions were performed for each formulation.

### 2.3. Physical Properties

#### 2.3.1. Density

The density of the samples was calculated by weighing the cylindrical samples and dividing their mass by their volume. A minimum of six repetitions was performed for each formulation.

#### 2.3.2. Water Uptake

Cylindrical samples (12 mm diameter, 4 mm height) were prepared by casting the hydrogel into silicone molds. One hour after application, the samples were weighed ($w_{i}$) using an analytical balance (Radwag, Radom, Poland, model no. AS 60/220.R2) and then immersed in DDW, covered, and placed in a 37 °C incubator. The samples were removed from the incubator after 1, 3, 5 and 24 h, blotted dry and weighed again ($w_{t}$). The water uptake was defined as the weight gained at each time point according to the equation:

(4) $$Water\;uptake\;\left(\%\right)=\frac{w_{t}-w_{i}}{w_{i}}\times 100$$

At least three repetitions were performed for each formulation.

#### 2.3.3. Crosslinking Degree

Crosslinking density was tested using a ninhydrin assay to determine whether the various crosslinker concentrations affect the relative amount of crosslinked and free amino groups. The results are described as the percentage of crosslinked amino groups relative to a formulation with no crosslinker (100% free groups). 

Hydrogel samples were lyophilized to remove any water, and 3 mg samples were prepared. The samples were then placed in a tube with 2 mL DDW, and ninhydrin reagent was added. The mixtures were placed in boiling water for 10 min and then allowed to cool to room temperature, after which 5 mL of 70% ethanol was added to each tube. Absorbance was read at 570 nm, and the results were compared to a calibration curve based on glycine as a reference material [27,28]. 

#### 2.3.4. Gelation Time

Gelation time indicates the working time available before the liquid solutions crosslink to become a hydrogel. A volume of 1 mL of hydrogel was injected into a well that contained a freely spinning magnet. The time required for the magnetic stirrer to stop spinning after mixing the 2 solutions together was defined as the gelation time. At least five repetitions were performed for each formulation.

### 2.4. Cell Viability Evaluation

In vitro cell viability tests were performed on mouse preadipocytes using an indirect method based on ISO 10993:12 (parts 5 and 12) for the biological evaluation of medical devices [29]. The hydrogels used for this study were non-foamed because following the extract liquid volume guidelines for indirect cytotoxicity tests (Table 1 in part 12 of ISO 10993 [29]) using a foamed hydrogel would result in using less hydrogel by weight per ml extract medium. Therefore, our test is considered the “safest” one; i.e., we chose to study the non-foamed formulations because they contain the highest EDC (crosslinker) and polymer contents of the studied system. The formulations tested are combinations of 200 mg/mL gelatin, 10 or 20 mg/mL alginate and 10 or 20 mg/mL EDC. Cells incubated in fresh medium were used as a control. 

The protocol was based on the requirements in ISO 10993 and was as follows: Cells were seeded into four 96-well plates, 5000 cells per well, with 0.2 mL fresh culture medium (Modified Eagle’s Medium (MEM) supplemented with 10% fetal bovine serum, 1% L-glutamine and 1% penicillin–streptomycin–nystatin). The plates were then incubated for 24 h in a humidified 37 °C, 5% CO_2_ environment. Extracts were prepared by immersion of hydrogel samples in cell culture medium and incubated for 24 h under the same conditions. Following the 24 h incubation, the cell culture medium was removed from the plates and replaced with a mixture of 30% extract in fresh medium or just fresh medium in the control group. This was done to imitate in vivo fluid replacement. The cells were incubated for a further 24 or 48 h, after which their viability was tested using the AlamarBlue™ assay (Invitrogen™, Rhenium, Modi’in, Israel).

### 2.5. Statistical Analysis

All data were processed using Excel (2010). Statistical analysis was performed using ANOVA (Tukey Kramer post hoc) via IBM SPSS (v. 27). A *p* < 0.05 was considered statistically significant and is indicated in the figures with an asterisk.

## 3. Results

The effect of the formulation parameters (alginate concentration, EDC concentration and polymer–air foaming ratio) on the mechanical–physical properties was studied, with emphasis on specific selected properties relevant to breast reconstruction. Cell viability was studied using mouse preadipocytes. 

**Figure 2:**
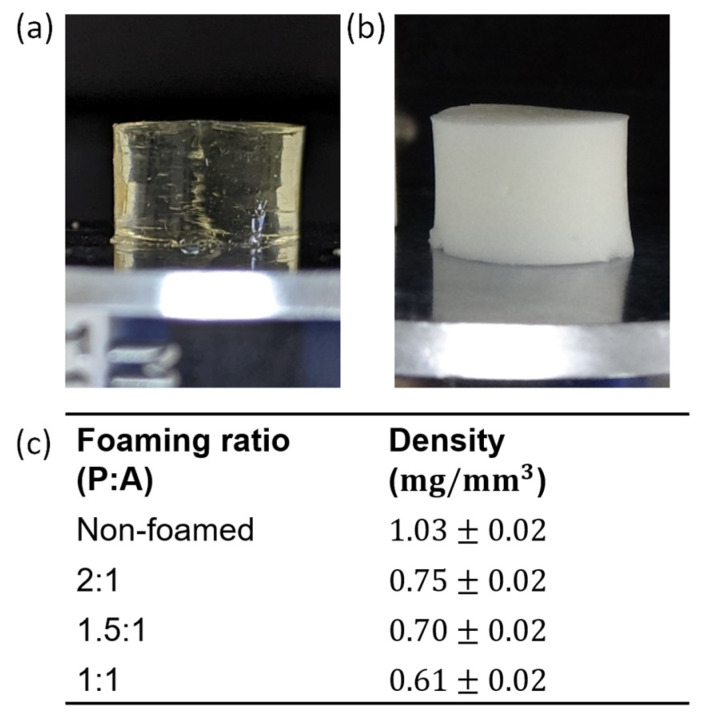
Hydrogel disks based on 200 mg/mL gelatin, 10 mg/mL alginate and 10 mg/mL EDC: (**a**) non-foamed and (**b**) foamed with a 1:1 (P:A) ratio. (**c**) The density of the polymer samples as affected by their foaming ratio.

Examples of the appearance of hydrogels prepared in the molds used for the compression and resilience tests are presented in Figure 2, along with the densities measured for each foaming ratio. The non-foamed hydrogel is transparent (Figure 2a), while the foamed hydrogels are opaque (Figure 2b) due to the presence of air bubbles. The density of the hydrogels ranges from 0.61 to 1.03 mg/mm^3^. As expected, the density decreases with the increase in the relative air content (Figure 2c). The alginate and EDC concentrations did not affect the appearance or density of the gels.

### 3.1. Mechanical Properties

#### 3.1.1. Compression

**Figure 3:**
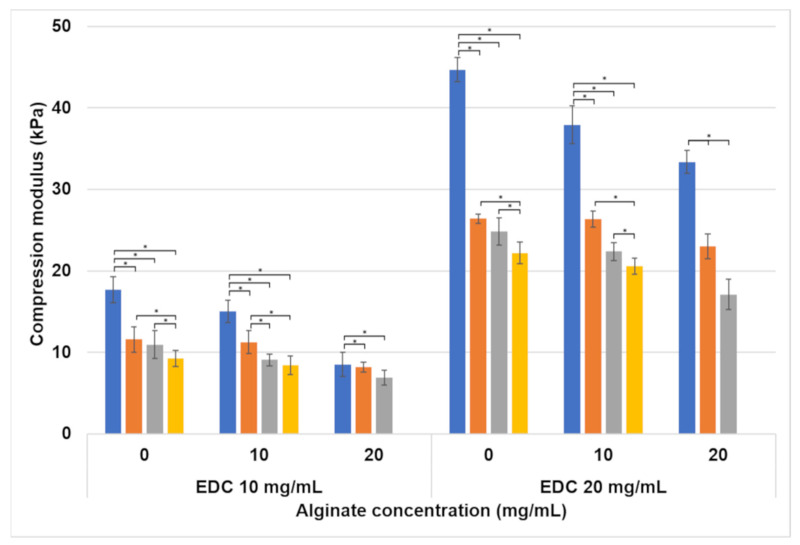
Compression modulus of hydrogel cylinders as affected by the alginate and EDC concentrations, and foaming ratio (polymer–air). Foaming ratios are presented as different bar colors: ■ non-foamed; ■ 2:1; ■ 1.5:1; ■ 1:1. Statistically significant differences are marked with *.

The compression modulus of the various hydrogels is presented in Figure 3. The lowest compression modulus (6.88±0.89 kPa) was obtained for the formulation containing the highest alginate concentration (20 mg/mL) and lowest EDC concentration (10 mg/mL) with a 1.5:1 (P:A) foaming ratio. The highest compression modulus (44.69±1.48 kPa) was achieved by a non-foamed hydrogel that has no alginate and 20 mg/mL EDC. The compression modulus generally increased with the increase in EDC concentration and decrease in alginate concentration. The foaming ratio also affected the compression modulus. When polymer–air ratios increase, less air is incorporated into the hydrogel. This results in a higher compression modulus. A very large increase in the modulus was obtained for the non-foamed 20 mg/mL EDC formulations compared to all other formulations. Our results show that the compression modulus is strongly affected by the EDC concentration. The polymer–air ratio and alginate concentration also affect the modulus but to a lesser degree.

**Figure 4:**
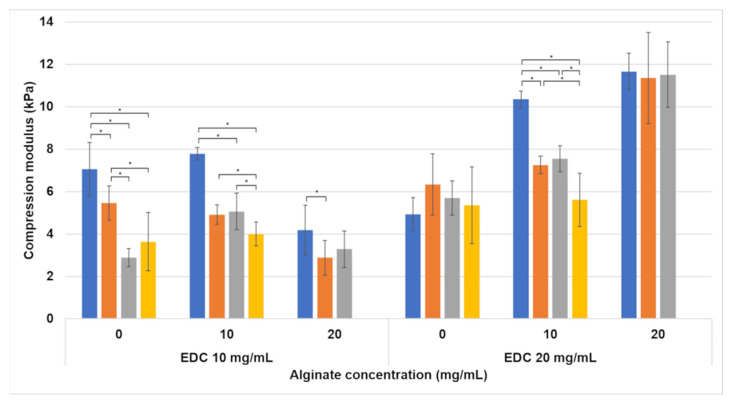
Compression modulus of hydrogel cylinders after immersion in water for 24 h at 37 °C. Foaming ratios (polymer–air) are presented as different bar colors: ■ non-foamed; ■ 2:1; ■ 1.5:1; ■ 1:1. Statistically significant differences are marked with *.

We tested the compression modulus of the hydrogels after swelling to evaluate the mechanical properties in water (Figure 4). Two things happen as the hydrogel swells—the matrix absorbs water and swells, and the pores fill with water. The water uptake into the matrix loosens the 3D network of crosslinks, thus decreasing the hydrogel’s stiffness. The lowest compression modulus is now 2.87±0.81 kPa for a hydrogel containing 20 mg/mL alginate and 10 mg/mL EDC, with a 1.5:1 (P:A) foaming ratio. The highest modulus (11.6 kPa) was obtained for the non-foamed hydrogel containing 20 mg/mL alginate and 20 mg/mL EDC. All compression modulus values decreased significantly after water uptake (*p* < 0.001). The sharpest decrease after water uptake was observed in the mechanical properties of formulations with no alginate. The general trend of increase in the compression modulus with the increase in EDC content and foaming ratio was preserved. However, the differences between the different foaming ratios were no longer as significant as before immersion in water. It is important to note that while the trend of lower modulus with an increase in alginate concentration was preserved for 10 mg/mL EDC samples, the 20 mg/mL EDC samples showed an opposite trend. 

#### 3.1.2. Tension

**Figure 5:**
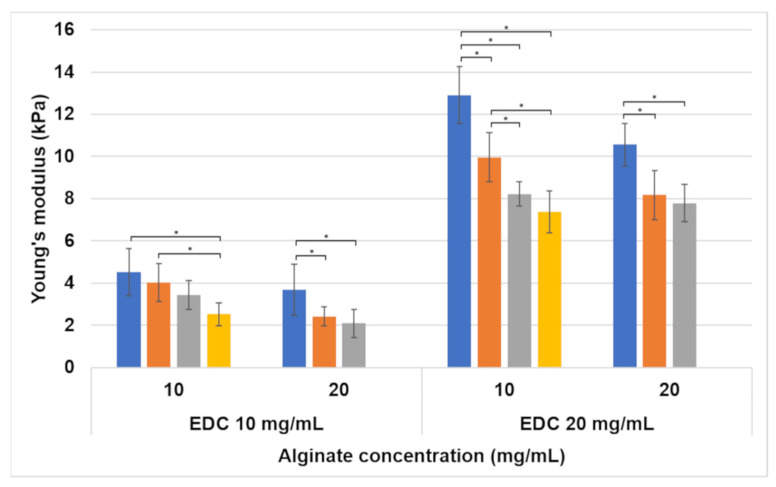
Young’s modulus of dog bone-shaped hydrogels tested in tension. Foaming ratios (polymer–air) are presented as different bar colors: ■ non-foamed; ■ 2:1; ■ 1.5:1; ■ 1:1. Statistically significant differences are marked with *.

The Young’s modulus of hydrogels tested in tension is presented in Figure 5. In general, increasing the EDC concentration results in a significant increase in the Young’s modulus. The alginate concentration and foaming ratio also affect the modulus, but their effects are less prominent. Increasing the foaming ratio or decreasing the alginate concentration results in an increase in the Young’s modulus. These effects are similar to those observed for compression (Figure 3). 

#### 3.1.3. Resilience

**Figure 6:**
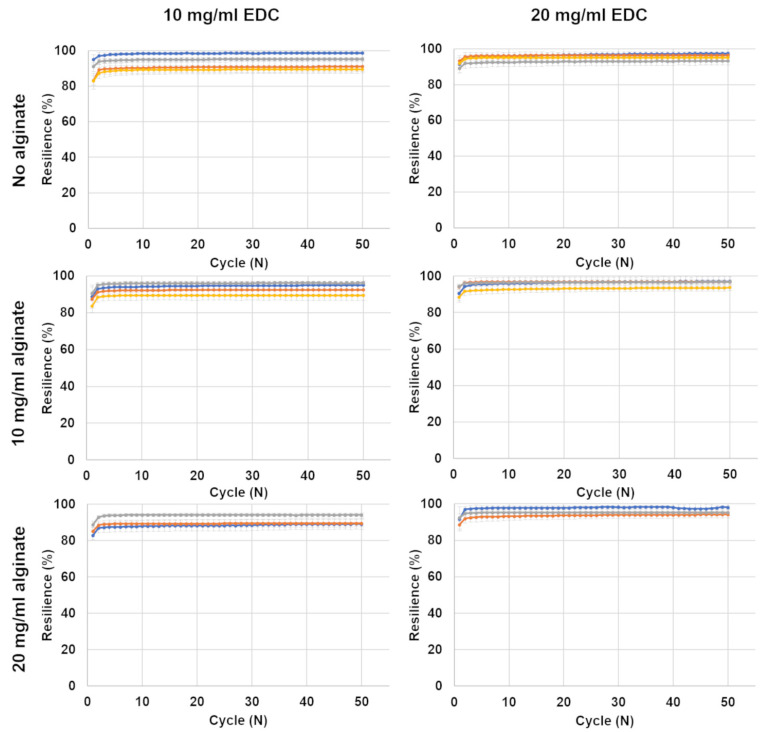
Resilience in compression of hydrogel cylinder samples. The samples were compressed to 40% strain and released repetitively for 50 cycles. Resilience is calculated by the energy preserved between cycles. EDC concentration is noted at the top of the figure, and alginate concentration on the left. Foaming ratios (polymer–air) are presented as different colors: ● non-foamed; ● 2:1; ● 1.5:1; ● 1:1.

Figure 6 shows the resilience calculated according to Equation (3) during each of the 50 compression cycles. After the first few cycles, the resilience remained almost constant for the duration of testing. The slight decrease is similar to preconditioning that is sometimes performed during testing [30], and it is common to not report the initial cycles that are reported here. All samples showed resilience of at least 88%, and most exhibited a resilience higher than 93%, except for the following formulations, all with 10 mg/mL EDC: no alginate with foaming ratios of 2:1 (P:A) and 1:1 (P:A), 10 mg/mL alginate with a foaming ratio of 1:1 (P:A) and 20 mg/mL alginate non-foamed and with a foaming ratio of 2:1 (P:A). The 20 mg/mL EDC hydrogels generally showed slightly higher resilience than the 10 mg/mL EDC formulations. Hydrogels with a foaming ratio of 1:1 (P:A) had a slightly lower resilience than the others.

### 3.2. Physical Properties

#### 3.2.1. Water Uptake

**Figure 7:**
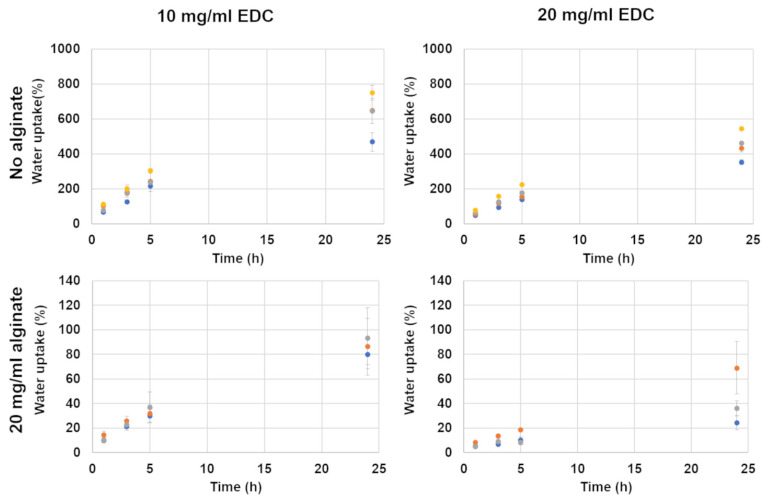
Weight gain as a measure of water uptake of hydrogels with different alginate and EDC concentrations. EDC concentration is noted at the top of the figure and alginate concentration on the left. Foaming ratios (polymer–air) are presented as different colors: ● non-foamed; ● 2:1; ● 1.5:1; ● 1:1. Please note the different y-axis scales in each graph.

The water uptake (percentage weight gained by the hydrogel due to water adsorption) of all studied formulations after 1, 3, 5 and 24 h in water is presented in Figure 7. Samples that did not include alginate exhibited water uptake of 200–250% after 5 h of incubation and 450–750% after 24 h of incubation when crosslinked with 10 mg/mL EDC. A similar trend was obtained for 20 mg/mL EDC, which exhibited water uptake of 150–220% after 5 h of incubation and 350–550% after 24 h. The addition of alginate, even at relatively low concentrations (20 mg/mL), resulted in a sharp decrease in water uptake for both types of formulations loaded with 10 and 20 mg/mL EDC. 

The lowest water uptake (10–20% after 5 h and 25–70% after 24 h) was obtained for samples loaded with 20 mg/mL alginate and 20 mg/mL EDC. Alginate incorporation thus strongly reduces the water uptake, and increasing the EDC concentration results in an additional slight decrease in water content. 

#### 3.2.2. Crosslinking Degree

**Figure 8:**
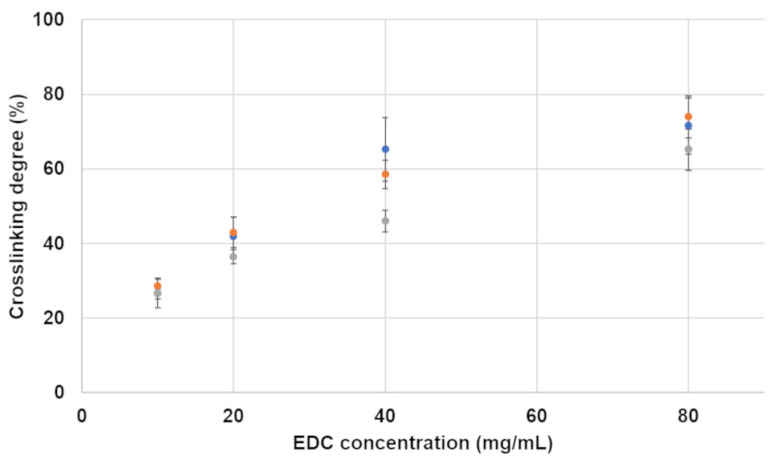
Crosslinking degree of hydrogels as affected by the crosslinker concentration. Only the non-foamed hydrogels are presented. The alginate concentrations are presented as different colors: ● no alginate; ● 10 mg/mL alginate; ● 20 mg/mL alginate.

The crosslinking degree, i.e., the percentage of crosslinked amino groups compared to a non-crosslinked sample, is presented in Figure 8. As expected, the crosslinking degree increased with the increase in the EDC concentration and reached a maximum of 74% for samples containing 80 mg/mL EDC. The alginate concentration apparently affects the crosslinking degree only slightly, as expected for the relatively low alginate concentration (10 or 20 mg/mL) compared to a gelatin concentration of 200 mg/mL, which was used in all studied formulations. It is important to note that crosslinking degrees of 27–29% and 36–43% were obtained for hydrogels containing EDC concentrations of 10 and 20 mg/mL, respectively.

#### 3.2.3. Gelation Time

**Figure 9:**
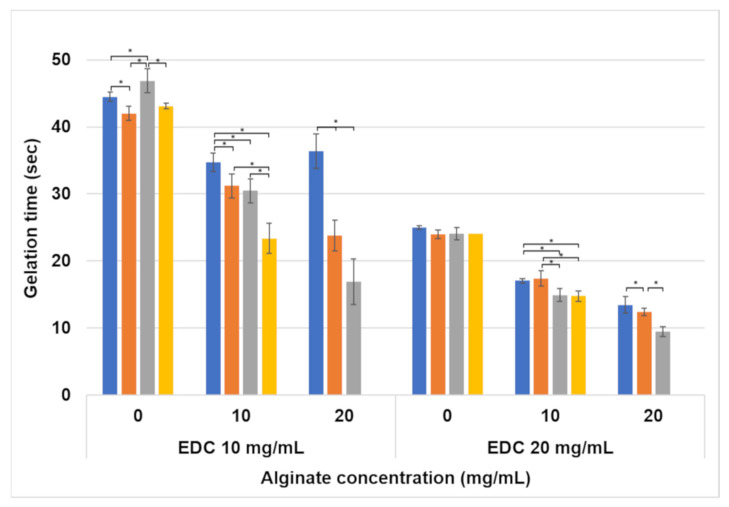
Gelation time of hydrogels as affected by the alginate and EDC concentrations, and foaming ratio (polymer–air). The foaming ratios are presented as different colors: ■ non-foamed; ■ 2:1; ■ 1.5:1; ■ 1:1. Statistically significant differences are marked with *.

Gelation time, i.e., the time required for the foamed polymeric solution to turn into a hydrogel, is important from an applicative perspective. This indicates the time available to the surgeon for molding the hydrogel into the desired shape after injection. A broad range of gelation times (9.5–47 s) was obtained for the studied formulations (Figure 9). The results show that higher concentrations of both alginate and EDC result in shorter gelation times. The foaming ratio has a minor effect on some of the formulations, in which the gelation time increases with an increase in the polymer–air ratio.

### 3.3. Cell Viability

**Figure 10:**
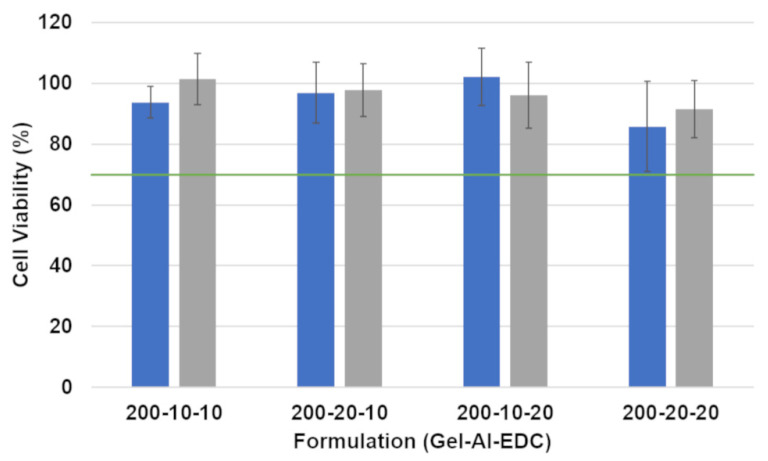
Effect of non-foamed hydrogels on the viability of preadipocytes. Viability is reported as a percentage of viability in control wells without any hydrogel. Blue and gray bars show the results after incubation of the cells in hydrogel extracts for 24 and 48 h, respectively. The green line marks 70% viability, which is considered by the FDA as the limit of cytotoxicity.

In addition to the above mechanical and physical tests, it is also very important to verify that the hydrogels do not induce toxic effects on cells. Indirect cytotoxicity tests of the hydrogels were, therefore, performed on preadipocytes using the AlamarBlue™ assay. The results are presented in Figure 10. According to the FDA (ISO 10993), formulations that result in more than 30% decrease in cell viability are considered cytotoxic. All hydrogel formulations exhibited over 80% cell viability, indicating that they do not induce a cytotoxic effect on preadipocytes. 

Only non-foamed hydrogels were tested because these have the highest density and, thus, the highest polymer content. Previous research conducted on similar hydrogels shows that cell viability is not affected by the foaming of the hydrogel, and similar results are obtained when testing foamed and non-foamed hydrogels [31].

## 4. Discussion

This study focused on the development and characterization of porous hydrogels for adipose tissue regeneration, in particular for filling defects created during partial mastectomies. We formulated hydrogels with varying polymer and crosslinker concentrations, as well as different porosities, in order to investigate the influence of composition and structure on the hydrogel properties. As expected, these variations in formulation significantly affected the hydrogels’ different mechanical–physical properties.

In compression, the effect of the formulation on the hydrogel is clear (Figure 3). An increase in porosity lowered the compression modulus. The decrease in modulus between non-foamed and all foaming ratios was significant, and the difference between foaming ratios was significant between most formulations (marked with *). This decrease was expected because the pores in the samples are essentially air pockets. Therefore, the higher the porosity, the lower the absolute polymer amount in the sample (Figure 2c). When the sample is compressed, these air pockets do not resist the load, and the hydrogel’s compression modulus decreases.

Increasing the crosslinker concentration from 10 to 20 mg/mL increased the compression modulus significantly. This happens because the crosslinker concentration is the limiting factor in the crosslinking reaction (Figure 8). An EDC molecule activates the carboxylic groups in both the alginate and the gelatin, which enables them to react with the amine groups in gelatin. Two types of crosslinking reactions are possible: gelatin–gelatin and gelatin–alginate. In both cases, the addition of a higher concentration of EDC increases the number of crosslinks in the hydrogel, and the higher crosslinking density leads to a denser 3D polymeric network. This increases the hydrogel’s stiffness. This effect is also seen in the compression after water uptake (Figure 4), with the exception of the formulations without alginate. In formulations containing 10 and 20 mg/mL alginate, the higher EDC concentration leads to a higher compression modulus, as seen in the non-swelled samples. This is not the case in formulations without alginate. This is probably caused by the different volume of water absorbed by the hydrogels. As can be seen in Figure 7, the water uptake of formulations without alginate is up to ten times higher than in formulations with alginate. According to a molecular dynamics simulation conducted in one of our previous studies, this is due to the fact that gelatin has a higher water solubility than the gelatin–alginate conjugate since it forms more hydrogen bonds with water molecules [32]. When comparing a constant hydrogel volume, the swollen sample will have fewer crosslinked units per volume than the non-swelled sample. This will lead to a sparse network that exhibits a low compression modulus. When the two opposite factors—high crosslinking density and high swelling degree—are taken into account, the very high swelling degree is dominant. 

The swelling effect on the compression modulus in the 10 and 20 mg/mL alginate samples was less pronounced than in the samples without alginate because they swell much less. However, there is still a significant decrease in the compression modulus and an increase in matrix flexibility with swelling. According to the tension–compression asymmetry, the tensile modulus is lower than the compression modulus even at low strains [33,34]. This brings the 10 mg/mL EDC samples into the range favorable for adipose tissue regeneration, not only in compression but also in tension. This was confirmed by the results presented in Figure 5, as hydrogels containing 10 mg/mL EDC range between a Young’s modulus of 2.1 and 4.5 kPa, depending on the alginate concentration and foaming ratio.

Alginate has an effect not only on the swelling degree but also on the compression modulus and, as mentioned above, on the Young’s modulus. These decrease with the increase in alginate concentration. This may be due to the competition for crosslinking sites, which increases with the increase in alginate concentration. When alginate is added to the gelatin–EDC crosslinking reaction, its carboxylic groups compete with the gelatin’s carboxylic groups for the gelatin’s available amine groups. It is possible that a less entangled network is created when there are more gelatin–alginate crosslinks rather than gelatin–gelatin crosslinks, and this decreases the hydrogel’s mechanical properties. When more EDC is added, in this case, 20 instead of 10 mg/mL EDC, the compression modulus increases for all formulations, which means that the polymers are found in excess and not the crosslinker. This was verified with the crosslinking degree assay shown in Figure 8. In the high EDC formulations, as more alginate is added, more polymeric chains remain non-crosslinked, decreasing the mechanical properties of the composite hydrogel.

An interesting observation made in Figure 4 is that the trend of a decrease in modulus with the increase in alginate is reversed after swelling in the 20 mg/mL EDC formulations. This reversal may be attributed to alginate’s significant reduction in swelling degree, which is further decreased by increasing the EDC concentration (Figure 7). As the matrix swells less, the reduction in compression modulus is also minimized. Perhaps the increase from 10 to 20 mg/mL EDC marks a critical point where the effect of the lower swelling degree surpasses the effect of the added alginate observed in the un-swelled samples.

Resilience is less clearly affected by the alginate ratio. It is affected to a greater extent by the polymeric–EDC ratio. Figure 6 shows the resilience results of all tested formulations divided by gelatin and alginate concentrations. The resilience of hydrogels containing 20 mg/mL alginate and 10 mg/mL EDC are slightly lower than those of the others (Figure 6). This may be attributed to the higher polymer concentration relative to the crosslinker concentration. Perhaps many polymer chains remain non-crosslinked, lowering resilience. The foamed hydrogels with a 1:1 (P:A) ratio generally had a lower resilience than the others, probably due to the lower density and smaller amount of polymer in the sample. The resilience of all formulations is considered high, and no decrease in resilience was observed as the cycles progressed. This will enable the use of these hydrogels in tissues that undergo cyclic deformations, such as those that breasts undergo when walking and jumping.

From a more applicative perspective, gelation time is an important parameter for the usability of the hydrogel. There must be enough time for the scaffold to be injected and molded before it crosslinks, while on the other hand, it must not take too long so that it will not disperse to a larger area than required. Gelation time is affected by both the polymer and crosslinker concentrations and less by the foaming ratios, as can be seen in Figure 9. The test that was performed measures the time it takes a magnet to stop spinning after the application of the hydrogel. This is an indirect measure of the hydrogel’s viscosity. The higher the crosslinker concentration, the faster the crosslinking rate and the increase in viscosity. Additionally, alginate creates a more viscous solution when added to gelatin [35], such that it takes less time for the polymer to reach a viscosity that stops the magnet from spinning. 

In fact, this test measures the time it takes to crosslink the solution. Since the viscosity of the solution increases during the crosslinking reaction, the magnet stops spinning when enough crosslinking occurs. Furthermore, since stirring the solution contributes to a more effective blending of the two solutions compared to the mixing process by the static mixer of the double-barrel syringe, it can be assumed that the actual gelation times in clinical practice will probably be longer than the times measured using this method. The clinician will thus have more time to shape the material after injection.

In a previous study [35], the rheological properties of similar hydrogels were examined, demonstrating the injectability of the hydrogels reported in this paper. The findings here indicate that the foaming ratio has only a minor effect on the viscosity of the solutions, and consequently, it also has a minimal effect on injectability.

Finally, but highly important, Figure 10 shows that none of the formulations had any cytotoxic effects on preadipocytes. EDC is a zero-length crosslinker that has been reported to be less toxic than other conventional crosslinking agents, such as formaldehyde and glutaraldehyde [36]. Fat tissue needs to grow into the scaffolds to restore the breast’s shape after a partial mastectomy. These results show that the combination of the chosen polymers and crosslinker is biocompatible and can be used safely with local cells. Our novel scaffolds are thus expected to enable the cells to thrive in the path to creating de novo tissue in vivo and filling the lumpectomy defect.

## 5. Conclusions

The studied hydrogels demonstrated a combination of elasticity and high resilience, two important factors for soft tissue regeneration. The mechanical and physical properties of the injectable hydrogels described in this study fit the requirements for adipose tissue growth while being based entirely on natural polymers. 

Our results show that most of the studied formulations exhibited resilience of at least 93% during 50 cycles, indicating that our new, highly porous structures are very resilient. Both the compression and Young’s modulus values increased when the EDC concentration was increased due to the increased degree of crosslinking. When increasing the polymer–air ratio, the moduli increased as well, while an increase in the alginate concentration only slightly decreased these moduli. Remarkably, these effects occur without compromising the hydrogel’s resilience. The gelation time and water uptake are affected mainly by the alginate concentration, where an increase in the alginate concentration results in a decrease in these physical properties. The gelation time is also strongly affected by the EDC concentration, while the polymer–air ratio has only a small effect on the physical properties.

The mechanical and physical characterization of the hydrogels presented here offers a variety of formulations suitable for adipose tissue applications. Porous hydrogels composed of 200 mg/mL gelatin, 10 mg/mL alginate, and 10 mg/mL EDC have proven to be appropriate from both mechanical and physical perspectives. These formulations exhibit moduli comparable to native tissue and possess the high resilience necessary for scaffold functionality.

The high biocompatibility of natural polymers, demonstrated here by the high preadipocyte viability, along with the specific properties achieved using these hydrogels, will allow the filling of small defects by providing a scaffold for adipose tissue regrowth in vivo. Designing a scaffold that is injectable and, therefore, minimally invasive will benefit both patients and surgeons, as it will improve patients’ quality of life while reducing hospitalization costs by minimizing the need for repeat surgeries. Our in situ injectable hydrogels provide a platform that has the potential to improve small defect reconstruction in breast tissue and other very soft tissues.

## Figures and Tables

**Table 1:** The formulations used in the study. All formulations are based on a concentration of 200 mg/mL gelatin. Foaming ratio is presented as the volumetric polymer-to-air ratio (P:A).

Alginate Concentration (mg/mL)	EDC Concentration (mg/mL)	Foaming Ratio (P:A)
0	10	Non-foamed, 2:1, 1.5:1, 1:1
	20	Non-foamed, 2:1, 1.5:1, 1:1
10	10	Non-foamed, 2:1, 1.5:1, 1:1
	20	Non-foamed, 2:1, 1.5:1, 1:1
20	10	Non-foamed, 2:1, 1.5:1
	20	Non-foamed, 2:1, 1.5:1

## Data Availability

The original contributions presented in the study are included in the article, further inquiries can be directed to the corresponding author.
